# Addressing the unmet needs of patients with BRAF-mutated melanoma in Latin America: Expert perspective

**DOI:** 10.3389/fonc.2023.1032300

**Published:** 2023-03-14

**Authors:** Pamela Salman, Andreia Cristina de Melo, Mariana Rico-Restrepo, Jeronimo Rodriguez, Andrea Russi, Rafael Aron Schmerling, Angela Zambrano, Gabriela Cinat

**Affiliations:** ^1^ Oncology Department, Oncovida Cancer Center, Santiago, Chile; ^2^ Brazilian National Cancer Institute, Grupo Oncoclínicas, Rio de Janeiro, Brazil; ^3^ Public Health Department, Americas Health Foundation, Bogota, Colombia; ^4^ Centro Oncológico, Hospital Médica Sur, Mexico City, Mexico; ^5^ Departamento de Oncología, Hospital Universitario San Ignacio, Centro Javeriano de Oncología, Bogotá, Colombia; ^6^ Hospital do Coracao (HCor), Oncology Department, São Paulo, SP, Brazil; ^7^ Departamento de Oncología, Fundación Valle del Lili, Cali, Colombia; ^8^ Instituto de Oncología Ángel Roffo, Universidad de Buenos Aires, Fundación CIDEA, Buenos Aires, Argentina

**Keywords:** *BRAF-*mutated melanoma, Latin America, melanoma, V600E, access, health policy

## Abstract

Melanoma represents an increasing public health burden with extensive unmet needs in Latin America (LA). A mutation in the *BRAF* gene is present in approximately 50% of all melanomas in White populations and is a target of precision medicine, with the potential to dramatically improve patient outcomes. Thus, increased access to *BRAF* testing and therapy is LA must be explored. At a multi-day conference, a panel of Latin American experts in oncology and dermatology were provided with questions to address the barriers limiting access to testing for *BRAF* mutation in patients with melanoma in LA, who may be eligible for targeted therapy to improve their prognosis. During the conference, responses were discussed and edited until a consensus on addressing the barriers was achieved. Identified challenges included ignorance of *BRAF*-status implications, limited human and infrastructural resources, affordability and reimbursement, fragmented care delivery, pitfalls in the sample journey, and lack of local data. Despite the clear benefits of targeted therapies for *BRAF*-mutated melanoma in other regions, there is no clear path to prepare LA for a sustainable personalized medicine approach to this disease. Due to melanoma’s time-sensitive nature, LA must aim to provide early access to *BRAF* testing and consider mutational status within treatment decision making. To this end, recommendations are provided and include establishing multidisciplinary teams and melanoma referral centers and improving access to diagnosis and treatment.

## Introduction

Melanoma represents an increasing public health burden with extensive unmet needs in Latin America (LA). Globally, melanoma incidence has risen and accounts for most skin cancer-related mortality (1), with 324,635 new cases in 2020 and 57,000 deaths worldwide (2) Moreover, studies report that patients with skin melanoma in low-and-middle-income countries, such as those in LA, are more likely to present with advanced disease and have poorer survival when compared to high-income countries (3–5).

Nevertheless, the prognosis of patients with advanced melanoma has dramatically improved in recent years. Before immunotherapy and targeted therapy emerged, the average five-year survival for patients with stage IV melanoma was 2·3%, and the median survival was eight to ten months (6). More recently, the advent of precision medicine has leveraged the increased understanding of tumor biology and the immune system’s role in developing personalized cancer therapies. One target of precision medicine is the BRAF protein, encoded by *BRAF*.

*BRAF* is a potent oncogene, present in approximately 50% of all melanomas in the White population, that plays a critical role in the Ras-Raf-mitogen-activated protein kinase/extracellular signal-related kinase (MEK) cell-signaling pathway (7, 8). People with melanoma who possess *BRAF* gene variants exhibit distinctive clinical features, with particularly aggressive biological behavior; patients are often younger and have tumors in areas without chronic sun exposure, with superficial spreading or nodular histology, and have an increased nevus count (9, 10). Additionally, *BRAF-*mutated tumors are more likely to metastasize to the brain (11, 12).

Data regarding melanoma mutations primarily comes from high-income countries (HIC), likely due to greater access to testing. Unlike in Europe and the US (13), data on melanoma incidence in LA is scarce. The available reports are mainly based on hospital records or private institutions that do not represent the general population, likely leading to underestimating the burden of this disease in the region (3, 14, 15). Additionally, the available data regarding mutational status has not been collected prospectively, thus having limited accuracy. Therefore, increased epidemiologic and data collection efforts are necessary to characterize the different populations and perform improved clinicopathologic correlation studies.

Melanoma epidemiology and *BRAF*-mutation frequency are heterogeneous and affected by ethnicity. For instance, a meta-analysis comparing the incidence rates of Asians and Whites found 19.5% and 40.3%, respectively (9). *BRAF*-mutation prevalence in LA has been found to be lower than in predominantly White populations. This is potentially a result of higher proportions of indigenous heritage and higher rates of acral-lentiginous melanoma in LA. However, ethnic variations among and within countries in LA are wide (9), and prior genomic knowledge of country-specific populations key to mutation screenings is largely lacking.^7^

Still, a few studies have documented *BRAF-*related melanoma in LA. In a single-institution cohort of 459 patients with melanoma in Barretos, Brazil, 34% carried a *BRAF* mutation (16). Another cohort showed V600 *BRAF* mutations in around 40% of cases (17, 18). However, this data is from a private institution and may not represent the general population (17–19). In Argentina, *BRAF* V600 mutation was reported in 50·2% of 354 patients (20).. Most of this sample is from patients from either the city or province of Buenos Aires, where most residents are White. In Mexico, studies identified *BRAF* V600E variant frequencies in primary melanoma studies ranging from 6·4% (3/47 patients in Mexico City) (21) to 73·0% (24/33 in Northeast Mexico) (22). In Chile, there is a complete lack of epidemiologic data.

Patients with melanoma with *BRAF* V600E and V600K mutations respond to clinically available BRAF inhibitors. Targeted treatment for patients with *BRAF* melanoma has reversed the poor prognosis associated with this molecular alteration (10). The success of targeted therapies in *BRAF*-mutated melanoma has led to the recommendation that patients with advanced disease and at high relapse risk be screened for V600 mutations to help guide therapeutic decision making (23).

Despite these advances, unmet needs remain, especially in regions such as LA, where determining mutational status and access to timely diagnosis and treatment are challenging. This review discusses the unmet needs of patients with *BRAF-*mutated melanoma in LA, including molecular testing strategies for detecting the mutation and their appropriate use within the regional context. The content is from the literature and panelists’ experience and opinion. The challenges to providing adequate and effective diagnosis and treatment for patients with *BRAF*-mutated melanoma are discussed, and recommendations on overcoming these barriers will be provided.

## Methods

### Study design and panelists

Americas Health Foundation (AHF) identified seven experts in oncology and dermatology from Argentina, Brazil, Chile, Colombia, and Mexico who have published in *BRAF*-mutated, melanoma, or health economics since 2016. As it was not practical to gather panelists from all the countries in Latin America together for a conference, the panel was chosen to provide a perspective of oncologists and dermatologists from countries across Latin America. The panel convened for a three-day virtual meeting on October 26-29, 2021, to discuss the need for region-specific recommendations. To identify the panel, AHF conducted a literature review to identify scientists and clinicians from the above countries who have publications relating to *BRAF*-mutated melanoma since 2016. Augmenting this search, AHF contacted opinion leaders from LA’s medical field to corroborate the list of individuals who adequately represented the necessary fields of study. All the experts who attended the meeting are named authors of this paper. An AHF staff member moderated the discussion. The authors retain complete control over the content of the paper.

Search strategy AHF conducted a literature review using PubMed, MEDLINE, and EMBASE. The following search terms were used: “BRAF,” “melanoma treatment,” and “cancer,” in combination with “Latin America,” “Mexico,” “Colombia,” “Argentina,” “Brazil,” and “Chile,” “molecular testing,” from 01/01/2016 until 04/10/2021. The articles identified were in English, Portuguese, and Spanish. Particular attention was paid to identifying literature and research in LA.

AHF developed specific questions to address barriers limiting access to testing *BRAF* variants in LA and assigned one to each panel member (**Supplementary Table 1**). A written response to each question was drafted by individual panel members based on the literature review and personal expertise. Each narrative was reviewed and edited by the entire panel during the three-day conference through numerous rounds of discussion until a complete agreement was reached. For issues where there was disagreement among the panel, additional dialogues took place until all panel members agreed to the content included in this paper. The recommendations developed were based on the evidence gathered, expert opinion, and personal experience and were approved by the entire panel. After the conference, the final manuscript was distributed by email to the panel for review and approval.

### Role of the funding source

This manuscript was supported by an unrestricted grant given to AHF, a 501(c)3 nonprofit organization dedicated to improving health care throughout LA, by Novartis. The funder had no role in the study design, data collection, data analysis, interpretation, and writing of the report.

### *BRAF* mutation testing

#### Patient selection and timing

International clinical practice guidelines (CPG) suggest that *BRAF* mutation testing be mandatory in patients with stage III or stage IV melanoma and high-risk resected disease (24). When metastases occur, it is recommended to use the metastatic sample. If it is unavailable, the analyses may be performed on lymph node metastases or the primary tumor, as there is a high degree of concordance between the *BRAF* status of primary melanomas and their metastatic lesions (25).

In the appropriate clinical context, initiating reflex testing at an earlier stage (IIB, IIC) for patients with limited access to frequent visits and specialist care can prevent unnecessary delays in targeted BRAF/MEK inhibitor therapy (26). They may prove to have a similar benefit for *BRAF*-mutated melanoma, as earlier treatment initiation may improve clinical outcomes for patients.

#### Testing methods

Different methods for *BRAF* testing exist and can be considered based on their utility in screening, confirmatory, and reference testing (27). Testing decisions often depend upon available methods and infrastructure, specificity and sensitivity, and variable cost and access throughout the region (**Table 1**) (28). Primary cutaneous melanomas, metastatic lymph nodes, or radiologically detected lesions are fixed with formaldehyde and paraffin-embedded in blocks that can be used for testing (29). Essential to accurate molecular testing, the tissue sample journey is complicated and involves prefixation, fixation, and post-fixation processes. The specific procedures of each step must be optimized to achieve preservation and ensure a high-quality sample.

**Table 1:** Sensitivity, specificity, and use in LA clinical practice for common *BRAF* diagnostic techniques.

Diagnostic Technique	Sensitivity (%)	Specificity (%)	Limit of Detection(%)	Use in clinical practice
IHC	93-97	92-98	5	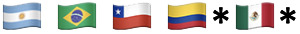
RT-PCR *(Cobas 4800 BRAF p.V600 and THxID -BRAF)*	98-100	98-100	0.5-5	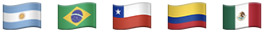
NGS	97.5	100	5	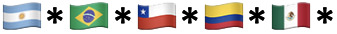
Sanger sequencing	92-98	100	20-25	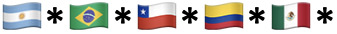
Pyrosequencing	>98	90-100	5-10	Only for research purposes
dPCR	100	95	0.001	Only for research purposes
MALDI-TOF MS	97.5	100	1-5	Only for research purposes

IHC, immunohistochemistry; RT-PCR, reverse transcription polymerase chain reaction, NGS, next generation sequencing; dPCR, digital polymerase chain reaction; MALDI-TOF MS, Matrix-assisted laser desorption/ionization- time of flight mass spectrometry.

* Only available in select highly specialized institutions.

**Figure 1:**
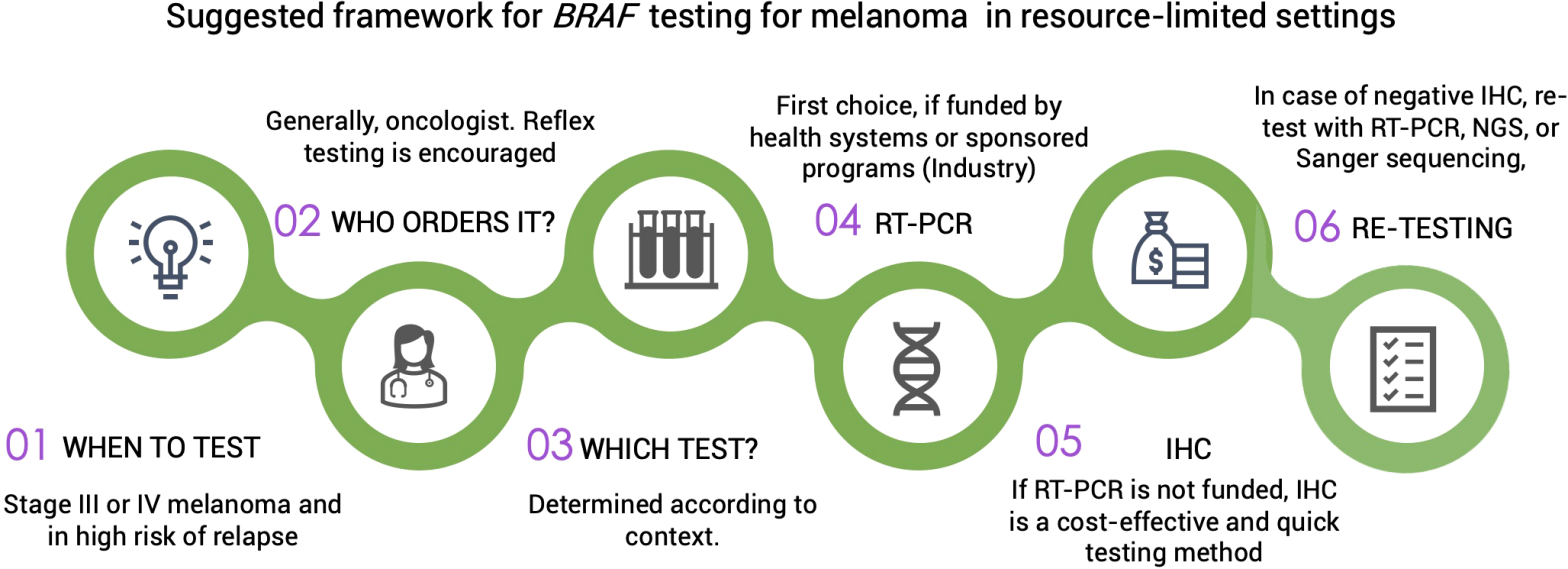
IHC, immunohistochemistry; RT-PCR, reverse transcription polymerase chain reaction; NGS, next generation sequencing.

Tumor heterogeneity in advanced-stage melanoma must be considered as it may have implications for molecular testing and, thus, treatment (30). To mitigate the risk of misinterpreting *BRAF* mutational status due to intratumor heterogeneity, testing should always be conducted on metastatic lesions when available (31, 32). Sequential analysis using confirmatory methods for detecting *BRAF* mutations is usually performed in high income countries (HIC). Nevertheless, this approach is not always feasible or cost-effective in limited-resource contexts. Ideally, confirmatory testing with real-time polymerase chain reaction (PCR), Sanger sequencing, or Next-Generation Sequencing (NGS) should be conducted in all patients with a negative test result through immunohistochemistry (IHC). Alternatively, a blood sample may be assayed for circulating tumor DNA, but its lower sensitivity compared with a tissue-based biopsy must be considered (33). **Figure 1** proposes a suggested pathway for *BRAF* testing in limited-resource settings.

##### Immunohistochemistry (IHC)

IHC is relatively simple and inexpensive, providing rapid results. *BRAF* V600E is the only variant that can be detected through this method (26, 34). Of note, IHC is not the best method regarding sensitivity: mutations may not be detected in some patients (31). However, employing IHC can be a cost-effective tool and a valuable supplement to conventional mutation testing to allow patients with V600E-variant metastatic melanoma to be triaged rapidly into appropriate treatment pathways (31, 32).

##### Real-time PCR-based techniques

Various real-time PCR-based methods are available, including Cobas 4800 and THxID-BRAF. Although these methods offer the advantages of a relatively quick turnaround time, they do not allow direct identification of the specific nucleotide sequence. These are commercially available companion diagnostic kits, each targeted to identify mainly V600E and V600K mutations (8).

##### Next-generation sequencing (NGS)

Despite NGS time, cost, and data-analysis demands, it can provide robust data on all *BRAF* and other actionable variants and quantify allele frequency. High specificity and sensitivity can be achieved with a small proportion of tumor DNA from the tissue sample. Its role is especially prominent in research, large mutation analysis, clinical trials, therapeutics, and confirmatory testing.

##### Sanger sequencing

Sanger sequencing, long known as the gold standard for reference testing in somatic variants, is 100% specific, providing quality sample preparation (8). It primarily serves as a confirmatory test in the case of inconclusive PCR methods, given its relatively higher costs and turnaround time (35).

##### Other methods

Several other methods for *BRAF* testing that offer high sensitivity and specificity exist but are generally not available in LA outside of the research context, primarily due to high costs and technological requirements. These methods include pyrosequencing, matrix-assisted laser desorption ionization time of flight mass spectrometry, allele-specific PCR, and droplet digital PCR.

### Systemic treatment for *BRAF*-mutated melanoma

Targeted therapy defied the conventional thinking in the United States, Europe, and Australia that patients in poor clinical condition due to advancing disease should not be treated. The uptake of these therapies throughout LA has been slower than in other regions due to access limitations, infrastructure issues, and cost constraints. Nevertheless, healthcare systems in the region could benefit from increasing the use of a precision medicine approach regarding patient outcomes, cost-effectiveness, and resource allocation.

Despite the fact that targeted therapy and immunotherapy have been highly effective in treating advanced *BRAF*-mutated melanoma (36–38), data comparing the two treatments revealed that targeted therapy has a greater response relative to immunotherapy. In contrast, immunotherapy confers longer-lasting results (39). Therefore, disease features, safety profiles, medical history, patient preferences, and access must be considered when making treatment decisions.

The first BRAF inhibitor developed, vemurafenib, surprised the oncological community with its Phase-I trial results showing rapid and profound responses that had never been seen in melanoma, though short-lived (40). The combination of MEK and BRAF inhibitors improved response rates, progression-free survival, and overall survival (OS) compared to monotherapy with BRAF inhibitors, as demonstrated with dabrafenib + trametinib (38); vemurafenib + cobimetinib (36); and encorafenib + binimetinib (37). For these three therapies, five-year progression-free and OS results were almost 20% and 35%, respectively. Some advanced melanoma characteristics associated with long-term responses to these drugs are normal lactate dehydrogenase (LDH) levels, less than three metastatic sites, and a good Eastern Cooperative Oncology Group performance status (38). These clinical features may help select patients with *BRAF*-mutated melanoma that would benefit most from targeted therapy in the long term. Patients with a high tumor burden, rapid progression, or poor performance status represent an unmet need for currently available drugs; reasonable access to target therapy for these patients is vital because of the rapidly progressing disease.

Combination therapy with immunotherapy (nivolumab/ipilimumab) followed by target therapy (dabrafenib/trametinib) is emerging as an option that may yield greater overall survival in patients with *BRAFV600*-mutated advanced melanoma (38, 39, 41, 42). The CheckMate 067 is a phase III trial which randomized previously untreated unresectable stage III or stage IV melanoma patients to receive nivolumab plus ipilimumab (four doses) followed by nivolumab; or nivolumab alone; or ipilimumab alone. The 6.5-year overall survival rates were respectively 57%, 43%, and 25% in patients with *BRAF*-mutant tumors and 46%, 42%, and 22% in those with *BRAF*-wild-type tumors, and the median overall survival is the longest in a phase III melanoma trial reported to date (43).

Few prospective data are available on sequential immunotherapy and BRAF/MEK inhibition for *BRAF*-mutant metastatic melanoma. The SECOMBIT is a noncomparative phase II trial which randomized patients with untreated, metastatic *BRAF*-mutant melanoma to receive A) encorafenib plus binimetinib until progressive disease followed by ipilimumab plus nivolumab; or B) ipilimumab plus nivolumab until progressive disease followed by encorafenib plus binimetinib; or C) encorafenib plus binimetinib for 8 weeks followed by ipilimumab plus nivolumab until progressive disease followed by encorafenib plus binimetinib. At a median follow-up of 32.2 months, the median overall survival was not reached in any arm. However, the 2 and 3-year OS rates showed that sequential immunotherapy and targeted therapy provide clinically meaningful survival benefits (44).

### Access to BRAF testing and treatment in LA

Although the proportion of patients with a melanoma diagnosis that undergoes *BRAF*-mutation testing in LA is unknown, it is likely lower than that of HIC due to restricted access. Access to diagnostic methods and treatments varies widely among and within countries in LA. Moreover, vast inequities exist in access between the regional private and public healthcare systems. Molecular testing demands an infrastructure comprising technological, financial, and human resources, which few institutions in LA possess (45). The complex technologies and processes are mostly only available in highly specialized centers, usually concentrated in major cities, leaving large populations underserved. In general, public hospitals do not offer this testing.

In countries in LA where targeted therapies are approved, *BRAF* testing is sometimes provided cost-free by the pharmaceutical industry, making it available to a large portion of the population for as long as the sponsored programs exist (45). Although this offers a short-term solution to access, it is not without problems. Logistical issues arise in the sample-handling journey, often resulting in diagnostic and treatment initiation delays. Further, these programs displace the government’s responsibility to provide reimbursement for testing, which is an undesirable situation.

Ideally, access to *BRAF* testing must be accompanied by access to targeted therapies. This is often not the case in LA, mainly due to economic constraints. Although at least one targeted therapy for *BRAF-*mutated melanoma is approved by regulatory agencies and available in many countries in LA, including Argentina, Brazil, Chile, Colombia, Mexico, and Uruguay, a lack of reimbursement, particularly within the public healthcare systems, continues to limit drug access (46). Of note, since advanced melanoma is a time-sensitive malignancy, access to both testing and therapy must be prompt.

## Discussion

### Challenges to *BRAF*-mutated melanoma management in LA

**Figure 2:**
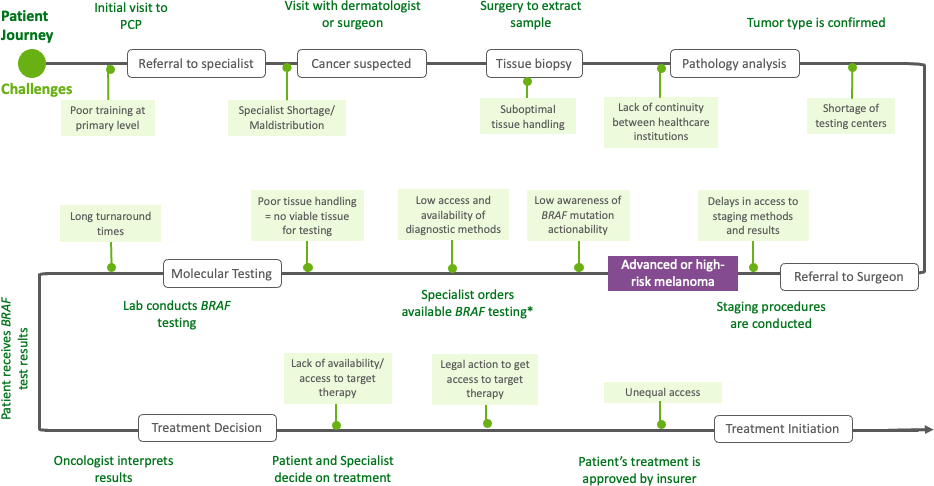
Patient journey of people with BRAF melanoma in Latin America. PCP, primary care physician.

A precision medicine approach to *BRAF-*mutated melanoma has demonstrated potential to improve health outcomes; however, factors inherent to precision medicine and LA’s healthcare systems create significant implementation obstacles. Given molecular pathology’s technical considerations and complexities, achieving precision medicine’s potential requires overcoming these hurdles. The barriers to quality management of *BRAF*-mutated melanoma in LA throughout the patient journey are depicted in **Figure 2**.

#### Affordability/Reimbursement

Precision medicine approaches demand high up-front investments in infrastructure, personnel training, and funding for molecular testing and targeted therapies. Although access to testing methods is not uncommon in the region, the corresponding targeted therapies are not always reimbursed or available. Additionally, anti-cancer drugs are proportionally much more expensive in LA when compared with higher income regions (47) because drugs are acquired with weaker currencies, and procurement strategies are not optimized. In the authors’ experience, the extent to which financing for testing limits access varies from not being an issue to affecting 75% of the time. The majority believe that financing for therapy is a limiting factor 25% of the time, although some report it is a factor 50-75% of the time.

#### Limited infrastructure

Healthcare centers with the capacity to provide melanoma care in LA are disproportionately located in major cities, leaving large areas underserved with unequal access levels. Even in major cities, pathology laboratories that meet the infrastructural demands of molecular tests used to diagnose *BRAF* mutations are scarce, resulting in long turnaround times for testing or unreliable test results.

#### Fragmented care

From diagnosis to treatment, melanoma care in LA is generally fragmented, a stark contrast to the current standard of care that involves a multidisciplinary team approach. Delays occur at virtually every step of the patient journey due to miscoordinations in care efforts at the primary level, during the diagnostic phase, and in treatment decision making. Tumor boards for melanoma, which can aid in overcoming diagnostic and management barriers, are not widely implemented.

#### Lack of human resources

There is a generalized shortage of medical personnel involved in melanoma care, including dermatologists, clinical oncologists, and pathologists, leading to high workloads and delays in diagnosis and treatment. This shortage may be related to the relatively small number of training opportunities for residencies and fellowships in these specialties within the region. Furthermore, a lack of resources in smaller cities or rural areas disincentivizes specialists from practicing in these areas, creating severe access gaps due to geographic resource maldistribution (48). At the primary care level, medical personnel lack training in identifying suspicious lesions and appropriate referral, which may also result in more advanced stages at diagnosis.

#### Lack of awareness of *BRAF*-status implications

Despite *BRAF* testing for advanced melanoma being compulsory based on unanimous international CPG, a generalized lack of awareness among stakeholders involved in melanoma decision making exists. Treating physicians do not always adhere to CPG, and similar treatment approaches are often taken for all or most patients with melanoma in LA, without regard to mutational status. Likewise, government, regulatory agencies, and payers do not always make evidence-based decisions to approve and reimburse targeted therapies and their corresponding diagnostic methods.

#### Pitfalls in the sample journey/quality assurance

Adequate institutional protocols and quality-control standards to regulate sample preparation are not standard across laboratories (49). Suboptimal practices within the tissue sample journey, including insufficient sample quantities, create technical challenges to *BRAF* testing. Because of limited infrastructural resources, there is a lack of continuity across the different healthcare institutions which the sample must pass in its journey, causing further delays.

#### Lack of local data

Region- and country-specific data to characterize the *BRAF*-variation prevalence in each population, enable accurate treatment decisions, and guide public policy are severely deficient. Global data corroborate the positive impact of introducing precision medicine strategies for melanoma treatment; however, the lack of local data hinders advancing this approach in the region. It is also necessary to define adequate metrics and indicators to track patient outcomes and determine the cost-effectiveness of targeted therapies for melanoma. Most decisions on healthcare resource allocation in LA are primarily based on cost. Yet coordinated (international) efforts to negotiate more accessible prices are not initiated by the governing bodies.

### Recommendations

This panel has addressed the lack of access to diagnosis and treatment for *BRAF*-mutated melanoma. With increasing health care costs and limited resources, a critical need exists to understand the root causes of these technologies’ underuse in the population for which they were developed. Additionally, efforts to increase access should be a collaborative, multi-stakeholder endeavor. The recommendations below address the challenges to widespread access to these diagnostic tools and, as a result, adequate treatments. Because these access issues are not exclusive to this region or this cancer, these recommendations may be tailored on a country-by-country and cancer-by-cancer basis.

1. **Improve affordability and reimbursement**
  Governments must work toward achieving a sustainable approach to sourcing high-cost cancer diagnostic methods and therapies as a region by:
  - Implementing procurement and contracting strategies such as managed entry agreements or risk-sharing strategies (50).
  - Leveraging negotiation power using pooled procurement, several countries can unite as a single buying bloc by combining their resources and requesting tests and doses.
  - Improving the coherence of approval and reimbursement for both pieces of the companion diagnostic (i.e., testing and therapy). Ideally, these regulatory pathways should provide an aligned channel for co-developed products to ensure innovation is not stifled.
2. **Improve testing infrastructure**
  Stakeholders must develop high-quality laboratories that can perform the molecular testing required for *BRAF* detection by:
  - Increasing investment in pathology departments and laboratories to meet the technological and training demands of high-quality molecular testing.
  - Establishing adequate quality-control standards, accreditation programs, and institutional protocols that regulate sample preparation and the quality of *BRAF* testing (51).
  - Creating centralized laboratories to perform the tests throughout the countries may help optimize turnaround times and save costs.
3. **Establish multidisciplinary teams and melanoma referral centers**
  Healthcare institutions should provide a *multidisciplinary approach* to melanoma management by:
  - Establishing multidisciplinary teams that include primary care physicians, dermatologists, clinical oncologists, surgeons, pathologists, geneticists, radiation oncologists, palliative care doctors, oncology nurses, and social workers.
  - Providing oncology navigators to guide and support patients through the medical and administrative process complexities related to cancer care.
  - Increasing referral centers for skin cancer care to promote care continuity and reduce delays (52–55).
4. **Raise awareness of melanoma and the actionability of *BRAF* variants**
  Medical societies and healthcare professionals must engage in continuous medical education at every level of care on the importance of determining *BRAF* mutational status.
  - Primary care physicians must be trained to recognize suspicious lesions and understand appropriate referral situations.
  - Specialists must understand the importance of determining *BRAF* mutations and facilitate diagnostic testing. Leveraging the concept of reflex testing may help reduce diagnostic delays.
  - Pathologists and molecular biologists must be adequately trained to conduct and interpret tests reliably and accurately.
5. **Increase BRAF testing**
  When indicated, healthcare professionals, medical societies, government, and patient organizations should promote testing for *BRAF* mutations.
  - All patients with metastatic or unresectable melanoma or those at high risk of relapse should be screened for *BRAF* V600 mutations, preferably in a metastatic lesion, and for adjuvant therapy in the primary tumor to guide therapeutic decision making (56).
6. **Address shortage and maldistribution of specialists**
  Governments, medical societies, and academic institutions must address the shortage and maldistribution of specialists by:
  - Increasing training opportunities in residency and fellowship programs in dermatology, surgical and clinical oncology, and pathology to address the lack of specialists in these fields.
  - Implementing virtual tumor boards or second opinion networks to bridge the gaps created by geographical disparities.
7. **Increase data collection and outcomes tracking**
  All stakeholders must prioritize funding for melanoma research to:
  - Establish national disease-specific registries to generate local data on which to base health policies tailored to the national context (50).
  - Define and quantify quality metrics, including indicators for access to diagnostic tools and therapies, to monitor patient outcomes and the impact of precision medicine strategies.

## Conclusion

There are wide opportunities in LA to improve the landscape of melanoma prevention, early detection, characterization, and management. Strategies to bolster primary and secondary prevention of skin melanomas must be prioritized as essential steps to improving the OS of patients with skin melanoma and also considering the economic implications this may have for health systems by avoiding disease evolution to more complex stages (57–60). That said, healthcare systems in LA must be better equipped to adapt to the complexities of the advanced stages of melanoma. Delaying diagnosis and treatment initiation negatively impacts progression-free survival and OS. In HIC, broad access to effective therapy for advanced disease has led to reductions in mortality. Similar results can be expected in LA if access is improved (55, 61). Despite the clear outcome benefits from targeted therapies for *BRAF*-mutated melanoma in other regions, there is no clear path to prepare LA for a sustainable personalized medicine approach to this disease. Due to melanoma’s time-sensitive nature, LA countries must provide early access to *BRAF* testing in appropriate situations and for mutational status to be considered within treatment decision making.

## Author contributions

PS and AM Writing-original draft, investigation, formal analysis, validation. MR-R Writing-review and editing, visualization, conceptualization, methodology, project administration. JR, AR, RS, AZ, and GC Writing-original draft, investigation, formal analysis, validation. All authors contributed to the article and approved the submitted version.
