# Supplementary material for: Addressing the unmet needs of patients with BRAF-mutated melanoma in Latin America: Expert perspective
Source: Front Oncol. 2023 Mar 14;13:1032300. doi: 10.3389/fonc.2023.1032300 (PMC10043339; doi:10.3389/fonc.2023.1032300)
Supplement: Supplementary file 1 [file Table_1.docx]

Supplementary Table 1. Questions given to panelists

1. **A)** What is the epidemiology and burden of disease of *BRAF* mutated melanoma in Latin America? **B)** Describe if, how, and why patients with *BRAF* mutations are different than other patients with melanoma, within the heterogeneity of the disease.
2. **A)** How are *BRAF*mutations diagnosed and at which point is testing usually ordered (e.g., before first-line treatment or at progression) in Latin America vs Global standards?

**B)** Who should request the test and act on the results? What are the roles of multidisciplinary teams and long term follow up for early-stage disease and how do these compare to Latin America’s reality?

**C)** Discuss the importance of molecular testing for accurate identification of mutation in *BRAF*, taking *BRAF* heterogeneity into consideration.  Is there an underdiagnosing of these mutations in melanoma patients in Latin America?

1. **A)** Which testing method is recommended for diagnosing BRAF mutation in melanoma (differences in sensitivity, trained personnel, tissue availability, costs)?

**B)** What are the barriers in the testing journey (patient journey/sample journey) and what can be done to overcome each one? (differences and challenges, physicians’ capabilities, access to testing, and the role of pathologists)

1. **A)** What is the value of biomarkers for optimal patient outcomes in BRAF + melanoma?

**B)** What are the challenges specifically to accessing molecular testing in LATAM and what are potential solutions? How can new biomarkers be incorporated into the testing journey?

**C)** Is there a precision medicine approach for melanoma patients in Latin America?

1. What considerations in terms of patient characteristics and clinical characteristics of BRAF + patients must be made in selecting the right patient to receive target therapy in BRAF-mutated melanoma and in which situations is it appropriate?
2. Please provide an overview of access and availability to targeted therapies for melanomas with *BRAF* mutations in Latin America. How do aspects such as reimbursement, infrastructure and human resources, availability of genomic data and research, and others impact access?
3. A) Considering the current and future trends in melanoma diagnosis and treatment and the evolving landscape, does Latin America need to prepare for a sustainable approach to personalized medicine in melanoma and treating the right patient with the right approach?

B) What effects on population health could be expected with more treatment alternatives available, sequencing and changing treatment paradigms along with more available evidence from clinical trials and real-world use in Latin America?

1. Considering the improvement in overall survival promoted by the adoption of targeted therapies for BRAF, what are some recommendations for its widespread adoption and to improve patient outcomes in Latin America? Specify the role played by HCPs to support optimal care for patients in Latin America.  Please provide a multistakeholder approach (HCPs/medical societies, payers, regulatory agencies, government).
